# Novel three‐dimensional ECG algorithm for reliable screening for cardiac amyloidosis

**DOI:** 10.1002/ehf2.15318

**Published:** 2025-05-04

**Authors:** Amir A. Mahabadi, Jan Knobeloch, Viktoria Backmann, Lars Michel, Markus S. Anker, Reza Wakili, Christian Fach, Stefan D. Anker, Tienush Rassaf

**Affiliations:** ^1^ West German Heart and Vascular Center, Department of Cardiology and Vascular Medicine University Hospital Essen Essen Germany; ^2^ INSOMNIA UG Weimar Germany; ^3^ Department of Cardiology (CBF), German Heart Center Charité University Medicine Berlin FU and HU Berlin Germany; ^4^ BIH Center for Regenerative Therapies (BCRT) Berlin Germany; ^5^ German Centre for Cardiovascular Research (DZHK) Partner Site Berlin Berlin Germany; ^6^ German Center for Cardiovascular Research DZHK, Partner Site Rhine‐Main Frankfurt am Main Germany; ^7^ Department of Medicine and Cardiology Goethe University Frankfurt Germany; ^8^ Kardiologie Schadowstraße Düsseldorf Germany; ^9^ Department of Cardiology (CVK) German Heart Center Charité Berlin Germany

**Keywords:** Cardiac amyloidosis, Electrocardiogram, Vector loop, Screening, Digital medicine

## Abstract

**Aims:**

Currently, there is no established screening tool for cardiac amyloidosis, leading to a delay in diagnosis in the majority of patients. We aimed to develop and validate a non‐invasive and easy to use tool that allows for screening of cardiac amyloidosis based on structured evaluation of three‐dimensional electrocardiograms (ECGs).

**Methods and results:**

We included patients with confirmed cardiac AL or ATTR amyloidosis and controls of patients with other cardiovascular diseases but without amyloidosis into two independent cohorts: a derivation and validation cohort. All patients received three‐dimensional ECGs and vector loops were categorized based on predefined patterns by two independent cardiologists. Consecutively, an AI algorithm was trained in the derivation cohort (*n* = 66 amyloidosis cases, *n* = 89 controls). This algorithm was then applied to the validation cohort (*n* = 33 amyloidosis cases, *n* = 67 controls).

Overall, 99 patients with amyloidosis and 156 controls were included (mean age: 69 ± 15 years, 79% male). In the derivation cohort, the AI algorithm reached a sensitivity of 85%, a specificity of 89%, a positive predictive value of 91%, and a negative predictive value of 87%. Applying the algorithm on the independent validation cohort, a sensitivity of 79%, specificity of 82%, a positive predictive value of 61%, and a negative predictive value of 92% was reached.

**Conclusions:**

We here describe a novel screening tool, which allows for reliable detection of cardiac amyloidosis.

## Graphical abstract

The present manuscript describes the derivation and validation of an algorithm for screening of cardiac amyloidosis using 3‐dimensional ECG. The algorithm is based on a ECG vector loop, acquired over the duration of 15 seconds using 4 electrodes. With this easy to perform method, we describe a high diagnostic accuracy for the detection of cardiac amyloidosis in a cohort of patients with severe heart failure.

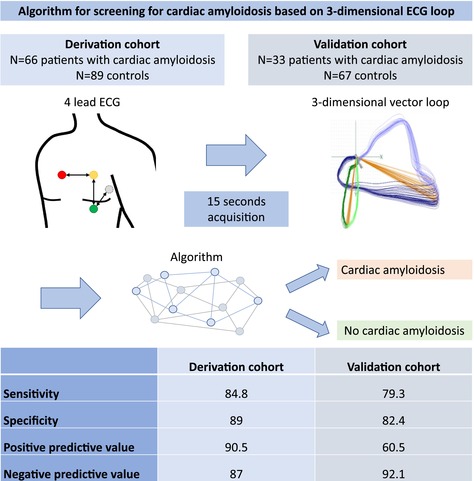

## Introduction

Cardiac amyloidosis, both due to amyloid‐transthyretin and amyloid light chain amyloidosis, is associated with a markedly increased morbidity and mortality.^1^ If diagnosed at early stages, specific therapies can preserve the patient's functional capacity and improve their outcome.^2^, ^3^, ^4^ In patients with high pretest probability for cardiac amyloidosis, algorithms are established in clinical routine for confirming the diagnosis.^5^ These include advanced cardiovascular imaging technologies such as cardiac magnet resonance imaging, radionucleotide imaging, or even invasive myocardial biopsy. Due to laborious nature, need for radiation exposure and limited availability of these established diagnostic tests, only a minority of patients are currently diagnosed in clinical routine,^6^, ^7^, ^8^ also due to the lack of broadly available screening tools for cardiac amyloidosis. Patients with cardiac amyloidosis frequently show specific electrocardiographic (ECG) characteristics.^9^ In addition to standard 12‐lead ECG assessment, three‐dimensional (3D) assessment of the ECG vector loop was shown to provide complemental information for evaluation of other cardiovascular diseases.^10^ We aimed to develop and validate a screening tool for cardiac amyloidosis based on structured evaluation of 3D ECGs.

## Methods

### Study cohort

We included patients from the West German Heart and Vascular Center with confirmed cardiac AL or ATTR amyloidosis as well as controls of patients with other cardiovascular diseases but without amyloidosis into two independent cohorts: a derivation and validation cohort. Patients with confirmed amyloidosis were selected from an ongoing prospective amyloidosis registry at our centre. Amyloidosis was confirmed either via myocardial biopsy or SPECT. Controls were selected from participants of the prospective ECAD II registry of patients with cardiovascular diseases, admitted to the West German Heart and Vascular Center. Control patients had either undergone myocardial biopsy for rule out of cardiac amyloidosis or no clinical signs of cardiac amyloidosis. For the derivation cohort, 66 patients with amyloidosis and 89 patients without amyloidosis were selected. For the validation cohort, a new set of 33 patients with confirmed amyloidosis and 67 controls were selected. The investigation was approved by the institutional ethics committee (amyloidosis patients: 19‐8806‐BO, controls: 21‐10066‐BO) and was performed in accordance with the Declaration of Helsinki. All patients provided informed consent.

### Clinical characteristics and covariate assessment

Information on traditional cardiovascular risk factors and comorbidities from the same presentation was drawn from the hospital information system. Arterial hypertension was defined as systolic blood pressure >140 mmHg, diastolic blood pressure >90 mmHg or on antihypertensive medication. Laboratory variables were assessed using standardized enzymatic methods. Hypercholesterinaemia was defined as an LDL cholesterol of >115 mg/dL or receiving lipid‐lowering therapy. Presence of coronary artery disease was defined as previous revascularization therapy (either stent or bypass surgery). Heart failure was defined according to current ESC guidelines.^11^ Standardized transthoracic echocardiography was performed in all patients. Left ventricular ejection fraction was assess by biplane Simpson's method. At least moderate valvular heart disease was defined according to current guidelines.^12^

### Three‐dimensional ECG assessment

**Figure 1:**
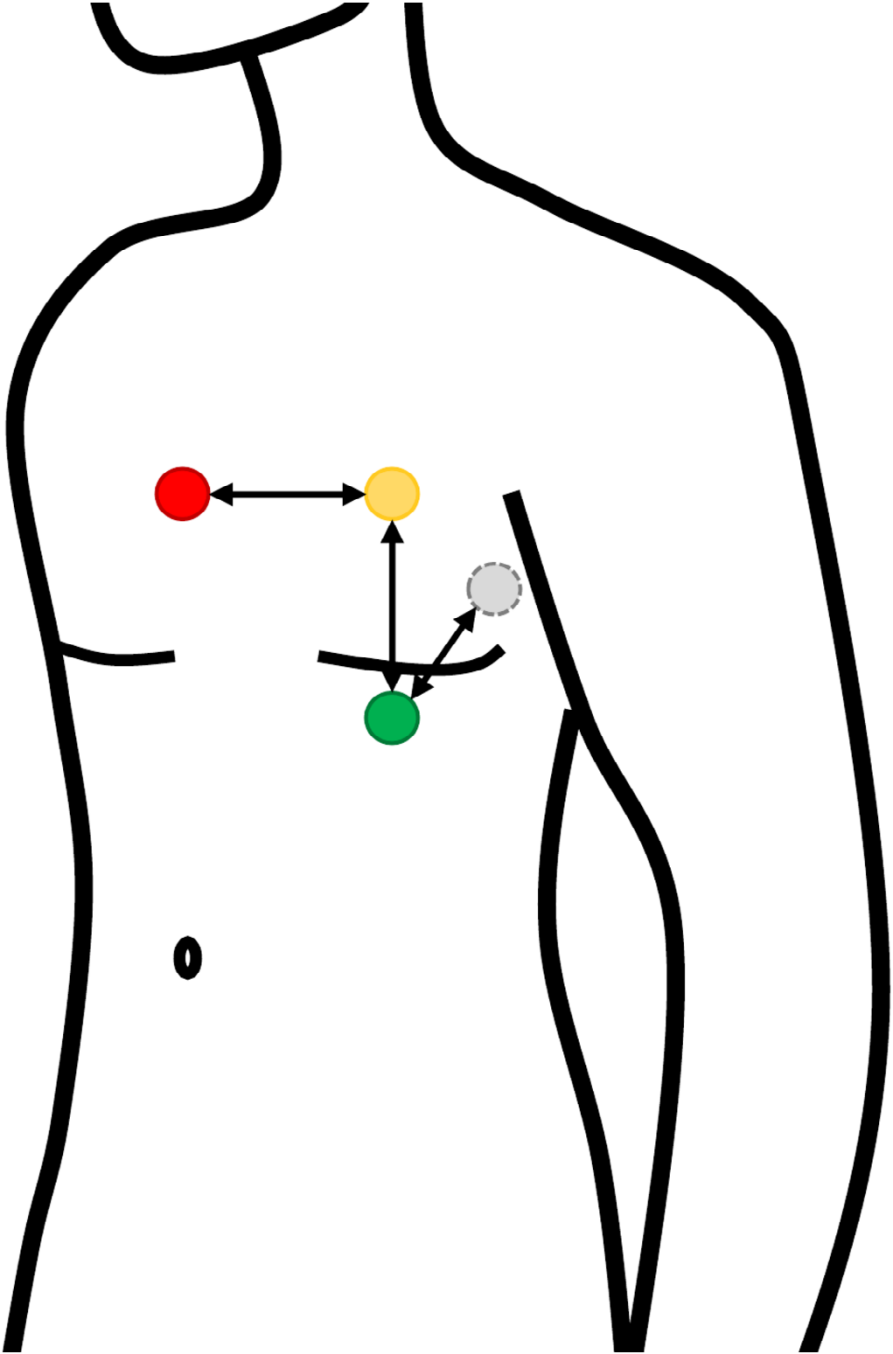
Positioning of the four leads of the ECG on the chest.

For acquisition of the 3D ECG vector loop, four electrodes were placed on the patient's chest in standardized manner. One electrode was placed in fifth intercostal space at the midclavicular line (equivalent to V4 from conventional 12‐lead ECG). The second electrode was placed at the corresponding position at the patient's back. Consecutively, the distance between the front and back electrode was measured. A third electrode was paced vertically above the first electrode with the distance depending on the distance from the first and second electrode. The forth electrode was placed right of the third electrode at identical height (*Figure* *1*). Patients were positioned in supine position and were requested to hold their breath. The 3D ECG vector loop was then recorded for 15 s.

### Algorithm development

#### Preprocessing

The electrode bonding scheme was used to obtain a spatial representation of the excitation of the heart. The result of a temporal measurement is a 3D ECG signal consisting of X, Y, and Z.^13^ This 3D ECG signal was processed and broken down to obtain the individual ECG complexes. The processing included signal filters to reduce interference, such as high‐frequency and low‐frequency influences. The determination of the individual QRS areas differed from the detection of the T‐areas. The QRS and T ranges were separated based on frequency using wavelet filter banks. As a rule, the QRS areas contain higher frequencies than T areas. Within the QRS range, the maximum vector R was determined as the relevant point. The T‐ranges were detected using a morphological analysis based on QRS. The relevant point of the maximum vector was also determined here.

**Figure 2:**
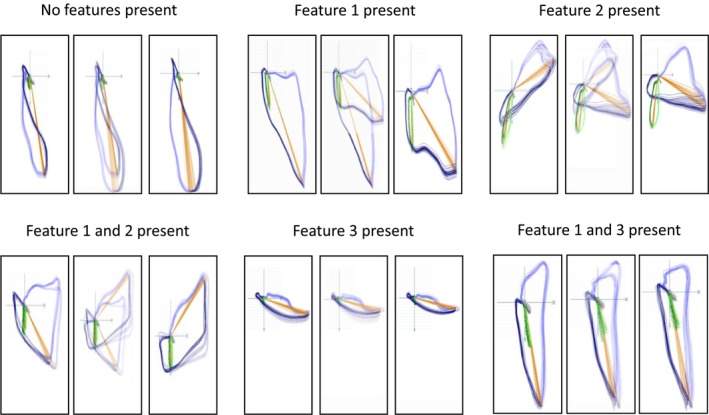
Schema for categorization of three‐dimensional ECG loops by experienced cardiologists prior to feature reduction. The three‐dimensional ECG loops were manually categorized into groups based on the shape of certain features. Via comparison of cases and controls, features were identified that were consecutively accentuated for feature extraction and algorithm development.

After preprocessing, the 3D ECG loops were manually categorized by two experienced cardiologists to detect potential differences between cases with amyloidosis and controls (*Figure* *2*). These features were consecutively accentuated for extraction and algorithm development.

#### Feature extraction

To analyse the presence of amyloidosis, the alignment of the QRS and T areas was determined using their maximum vectors. The alignment is not relevant in an absolute sense but is relevant in relation to the QRS and T areas. In a medically normal case, both must be approximately aligned. The angle between the two maximum vectors was determined for this purpose. Consecutively, shape analysis was performed using Fourier descriptors. For this purpose, the main plane of the loop in space was first determined using principal component analysis. This resulted in the principal plane of the loop in the first two principal components. The Fourier descriptors were then determined as features in this principal plane. This resulted in a reduction of the shape to the essential information. Furthermore, the eccentricity and compactness were determined with the help of the convex hull, the loop in the main plane.

#### Evaluation of the features

Different variants were created to determine the best combination of features. To differentiate between the medically normal signals and those with proven amyloidosis, a hyperplane was found that separates the positions defined by the feature vectors (the individual patients) in the multidimensional feature space. Features that were too similar or had the same information content were removed. In addition, we anticipated to detect a hyperplane that manages with a minimum number of dimensions in order to solve the problem adequately.

### Statistical analysis

The baseline characteristics are presented as the mean ± standard deviation or median (interquartile range) for continuous variables and as frequency and percentages for categorical variables. For the derivation and validation cohort, sensitivity, specificity, positive predictive value, and negative predictive value were calculated based on the assessment of cardiac amyloidosis of the algorithm with the clinically confirmed diagnosis of cardiac amyloidosis as gold standard. In sensitivity analysis, we stratified the cohort into patients aged above versus below median age. Differences in baseline characteristics between the groups were compared using the chi‐square test, a two‐sided unpaired *t*‐test, or the Mann–Whitney test, as applicable. Afterwards, we described the diagnostic performance for both age groups as well as stratifying by NAC stage (I vs. ≥II), combining the derivation and validation cohort. All analyses were performed using SAS software (version 9.4, SAS Institute Inc.). A *P* value <0.05 indicated statistical significance.

## Results

Overall, 255 patients (mean age: 69 ± 15 years, 79% male) were included in our analysis. Ninety‐nine patients had confirmed cardiac amyloidosis, whereas cardiac amyloidosis was ruled out in 156 patients. Patients with cardiac amyloidosis were older, were more frequently male, and had higher prevalence of atrial fibrillation and higher levels of NT‐proBNP (median NT‐proBNP 3446 vs. 1547 pg/ml for patients with vs. without cardiac amyloidosis). In contrast, patients without cardiac amyloidosis had lower left ventricular ejection fraction and higher prevalence of aortic valve stenosis. The majority of patients in both groups had previously been diagnosed with heart failure. Detailed patient characteristics are provided in *Table* *1*. Stratifying the cohort by patients above vs. below median age (77.4 years), we found that male sex, presence of coronary artery disease, atrial fibrillation, and hypercolesterinaemia was more frequent in older patients, while the distribution of age and sex for the younger amyloidosis patients was comparable with the controls.

**Table 1:** Baseline characteristics of patients without and with amyloidosis. For patients with amyloidosis, baseline characteristics are further stratified by patients aged above versus below median age (77.4 years)

	Patients without cardiac amyloidosis *N* = 156	Patients with cardiac amyloidosis *N* = 99	Patients with cardiac amyloidosis with age < median *N* = 49	Patients with cardiac amyloidosis with age ≥ median *N* = 50	p‐value for difference of patients aged < vs. ≥ median
Mean age, years	64 ± 17	75 ± 11	67.0 ± 10.4	82.1 ± 2.9	<0.0001
Female sex, %	24	17	28	8	0.009
Coronary artery disease, %	58	48	35	59	0.017
Atrial fibrillation, %	38	59	48	66	0.035
Arterial hypertension, %	70	67	60	73	0.18
Hypercholesterinaemia, %	75	73	59	86	0.002
At least moderate aortic valve stenosis, %	19	10	4	14	0.086
At least moderate mitral valve regurgitation, %	31	34	27	40	0.16
At least moderate tricuspid valve regurgitation, %	27	32	37	28	0.35
Other at least moderate valve disease, %	9	6	2	10	0.097
Mean LV ejection fraction, %	42 ± 15	49 ± 10	49.2 ± 11.8	48.7 ± 9.0	0.83
Heart failure, %	91	99	98	100	0.31
Hypertrophic cardiomyopathy, %	10	—	—	—	—
Dilated cardiomyopathy, %	12	—	—	—	—
NAC stage, %	—				0.07
I		49	38	60	
II		29	38	20	
III		6	12	0	
IV		16	12	20	
Left ventricular muscular mass index, g/m^2^	135 ± 50	171 ± 66	173 ± 71	168 ± 62	0.7
Intraventricular septum diameter in diastole, mm	1.19 ± 0.36	1.74 ± 0.45	1.82 ± 0.49	1.65 ± 0.37	0.04
Median NT‐proBNP values, pg/mL	1547 (567; 4383)	3446 (1258; 6772)	2831 (972; 6523)	3766 (1803; 6843)	0.20

In the derivation cohort, the algorithms reached a sensitivity of 85% and a specificity of 89%. Likewise, high positive and negative predictive values were achieved (*Table* *2*). For the validation cohort, we specifically included twofold more controls than amyloidosis cases to better adapt the cohort that would be applied to a potential screening tool in clinical routine. Due to the lower proportion of patients with cardiac amyloidosis, the positive predictive value slightly decreased as expected with approximately two out of three patients with positive 3D ECG screening having a diagnosis of cardiac amyloidosis. Sensitivity, specificity, and especially negative predictive value remained at values around 80–90% (*Table* *2*). Sensitivity, specificity, negative‐ and positive predictive value of the 3D ECG loop‐based algorithm were independent for the patient's age and NAC stage (*Table* *3*).

**Table 2:** Sensitivity, specificity, and positive and negative predictive value in the derivation and validation cohort

	Derivation cohort	Validation cohort
	*N* = 89 controls	*N* = 67 controls
	*N* = 66 amyloidosis cases	*N* = 33 amyloidosis cases
Sensitivity	84.8	79.3
Specificity	89	82.4
Positive predictive value	90.5	60.5
Negative predictive value	87	92.1

**Table 3:** Sensitivity, specificity, and positive and negative predictive value for amyloidosis patients stratified by median age and NAC stage (stage I vs. ≥II)

	Age groups		NAC stage	
	< median	≥ median	Stage I	Stage ≥II
Sensitivity	81.2	84.1	85.7	84.0
Specificity	88.4	84.4	89.0	89.0
Positive predictive value	82.1	75.3	70.0	70.0
Negative predictive value	87.4	90.6	95.2	94.5

## Discussion

We here describe the first development and evaluation of an algorithm for screening of cardiac amyloidosis based on the 3D ECG loop signal. We showed that parameters derived from the electric signal of the heart differentiate between patients with and without amyloidosis and when aggregated in an algorithm can reliably detect cardiac amyloidosis. The diagnostic accuracy of the algorithm was successfully validated in a second, independent cohort with especially high negative predictive value. If integrated into initial workup of appropriate patient cohorts, the described 3D ECG evaluation may allow for an easy and reliable screening of cardiac amyloidosis even in an ambulatory primary care setting.

Cardiac amyloidosis, either ATTR or AL cardiac amyloidosis, is a progressive cardiomyopathy leading to heart failure and markedly decreased prognosis.^14^ Over the last decade, tremendous improvements have been made for diagnosing and treating especially ATTR cardiac amyloidosis, with multiple other treatment strategies currently being evaluated in ongoing clinical trials.^2^, ^3^, ^14^, ^15^, ^16^, ^17^, ^18^, ^19^ Available data suggest that these therapies are most effective when applied at early stages of the disease as reflected by lower NYHA class or NT‐proBNP levels.^15^, ^17^, ^20^ This underlines the unmet need for a screening tool for cardiac amyloidosis that can be applied in ambulatory general cardiology services or even in primary healthcare settings.^21^ Among patients with non‐dilated hypertrophic ventricles with normal ejection fraction, the combination of low QRS voltages with interventricular septum ≥1.6 cm lead to a high sensitivity and specificity.^22^ We here describe such a tool that utilizes the electric signal of the heart and enables assessment within seconds. The fast application together with the low requirements on resources makes it cheap to perform without the need for radiation exposure or any other harm to the patient.

Algorithms based on artificial intelligence (AI) have led and most likely will further lead to major breakthroughs in cardiovascular medicine in the near future. Likewise, for detection of cardiac amyloidosis, several AI‐based evaluations have been described. In patients undergoing scintigraphy, AI‐based screening of cardiac amyloidosis‐suggestive uptake led to reliable detection of cardiac amyloidosis and reduced inter‐rater variability.^23^ Using two‐dimensional echocardiography as well as global longitudinal strain analyses, AI algorithms have been described for evaluation of presence of cardiac amyloidosis as well as patient's prognosis.^24^ Similar reports also exist for cardiac magnet resonance imaging.^25^ These all combine that they necessitate dedicated cardiac imaging with specified image acquisition, which availability is limited outside of specialized centres. The here described algorithm is based on a four‐lead ECG signal that can be acquired within seconds by assistance personnel with minimal training. It does not require any dedicated equipment, expertise, or other specialized resources. Besides the here described screening tool, AI algorithms have been developed for standard 12‐lead ECGs to detect cardiac amyloidosis with comparable diagnostic accuracy.^26^ However, for implementation of these into clinical routine, the algorithms would need to be implemented into existing ECG machines, limiting their fast distribution. In contrast, the here described algorithm allows for evaluation of the ECG signal via a mobile device or laptop via an app, implementing low barriers for its distribution into clinical practice.

### Clinical implications

**Figure 3:**
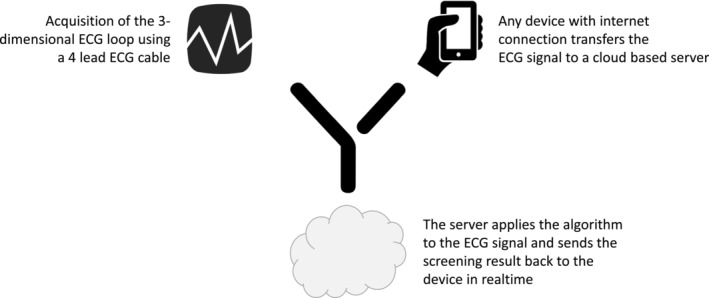
Potential implementation of the algorithm into clinical routine. The three‐dimensional ECG signal is acquired using a four‐lead ECG cable with USB port. This ECG cable is connected to a mobile device or personal computer, sending the signal to a server for evaluation using the described algorithm. The result can be displayed on the screen of the device within seconds.

Early detection of cardiac amyloidosis is essential for initiation of specific therapies to maintain patient's well‐being and improve the patient's prognosis. If pretest probability for the presence of cardiac amyloidosis is high, strategies are established in clinical routine to confirm the diagnosis.^5^ A majority of patients with cardiac amyloidosis are still undiagnosed with markedly higher prevalence being reported when systematic screening is applied.^27^, ^28^ However, all currently established tests for evaluation of cardiac amyloidosis such as scintigraphy or myocardial biopsy do not allow for a broader screening due to the necessity of radiation exposure, their invasive nature, and limited availability.^29^, ^30^, ^31^ The here described algorithm based on a 3D ECG loop acquired for 15 s may overcome the current lack in availability of broadly available screening tools. Appropriate ECG cables with USB connection are available on the marked at low costs. If connected to any device with Internet access (mobile phone, tablet, or laptop), an app can enable fast screening (*Figure* *3*). Using this approach, a fast and low‐cost screening for cardiac amyloidosis could be implemented in primary healthcare settings. The efficiency of the described model may further be increased in pre‐screened populations, for example, patients undergoing cardiac magnet resonance imaging suggestive of cardiac amyloidosis or patients with presence of ‘red flags’ of cardiac amyloidosis such as carpal tunnel syndrome or history of biceps tendon rupture. In addition, when transferred to larger databases, the applied methods may enable further refinement of the algorithm, leading to further improvement in its diagnostic accuracy.

### Study limitations

Our results are based on a single‐centre experience and a predominantly white cohort. External validations are needed to confirm that the algorithm enables detection of cardiac amyloidosis especially in other ethnic groups with similar accuracy in a multicentre setting. In addition, the overall sample size of our two cohorts is limited. While a total of 99 patients with cardiac amyloidosis were included in the development and validation cohorts, application to larger cohorts will likely enable further improvement of the algorithm in the future. In addition, patients with and without amyloidosis were not fully balanced with respect to age, sex, and history of concomitant cardiovascular diseases. However, also, the control group reflects a multi‐morbid heart failure cohort with a median NT‐proBNP level of approximately 1500 pg/mL, a cohort that may qualify as a potential target for amyloidosis screening. While differences in comorbidities may introduce biases, comparable diagnostic accuracy was observed in all subgroup analyses. Lastly, since not all control patients received myocardial biopsy for ruling out cardiac amyloidosis, we cannot rule out that some patients may have had early stages of cardiac amyloidosis in the control group. While no signs of cardiac amyloidosis were present in all control patients, this, however, may only have biased the results towards the null.

## Conclusions

We here describe the development and validation of an algorithm based on a 3D ECG loop signal that allows for the detection of cardiac amyloidosis with high diagnostic accuracy. This novel screening tool enables for easy and reliable detection of cardiac amyloidosis and could be broadly applied even in primary healthcare settings.

## Author contributions

We can assure that all authors have (1) provided conception and design or analysis and interpretation of data, or both; (2) been drafting of the manuscript or revising it critically for important intellectual content; and (3) provided final approval of the manuscript submitted.

## Conflict of interest

A.A.M. received honoraria, lecture fees, and/or grant support from Amgen, Daiichi‐Sankyo, Edwards Lifesciences, and Novartis, Sanofi, all unrelated to this work. T.R. received honoraria, lecture fees, and/or grant support from Edwards Lifesciences, AstraZeneca, Bayer, Novartis, Berlin Chemie, Daiichi‐Sankyo, Boehringer Ingelheim, Novo Nordisk, Cardiac Dimensions, and Pfizer, all unrelated to this work. R.W. received honoraria, lecture fees, and/or grant support from Boehringer‐Ingelheim, Bristol‐Myers‐Squibb, Daiichi‐Sankyo, Pfizer, Boston Scientific, Biotronik, Abiomed, Zoll, Novartis, Medtronic, Bayer, ArtiCure, and Abbott. L.M. received honoraria, lecture fees and/or grant support from Alnylam, AstraZeneca, Bayer, Bristol Myers Squibb, and IIFM e.V. S.D.A. received grants and personal fees from Vifor and Abbott Laboratories and personal fees for consultancies, trial committee work and/or lectures from Actimed, AstraZeneca, Bayer, Boehringer Ingelheim, Brahms, Cardiac Dimensions, Cardior, Cordio, CVRx, Cytokinetics, Edwards, Farraday Pharmaceuticals, GSK, Impulse Dynamics, Lilly, Mankind Pharma, Medtronic, Novartis, Novo Nordisk, Occlutech, Pfizer, Regeneron, Relaxera, Repairon, Scirent, Sensible Medical, Vectorious, and V‐Wave. He was named co‐inventor of two patent applications regarding MR‐proANP (DE 102007010834 and DE 102007022367), but he does not benefit personally from the related issued patents. A.A.M., J.K., R.W., C.F., and T.R. are co‐founders of Mycor GmbH, a company focusing on the development of AI‐based ECG‐algorithms.

## Funding

None.
